# Inappropriate Mixed Addition of Plant Residues Weakens the Remediation Effect of Crude Oil-Contaminated Soil

**DOI:** 10.3390/plants15152263

**Published:** 2026-07-24

**Authors:** Hongyan Hui, Liping Li, Xianyang Pan, Jing Xie, Xin Liu, Wenqing Jiang, Xincheng Huang, Xiaoxi Zhang, Bo Liu

**Affiliations:** 1 College of Life Sciences, Yan’an University, Yan’an 716000, China; hhy554999333@126.com (H.H.); llp07082024@163.com (L.L.); pxy126219@sina.com (X.P.); xiejing2190325@163.com (J.X.); liuxinn219@163.com (X.L.); jwq2026219@126.com (W.J.); hxc2026219@126.com (X.H.); 2 Key Laboratory for Applied Ecology of Loess Plateau, Shaanxi Province, Yan’an 716000, China

**Keywords:** crude soil contamination, necrophytoremediation, nonadditive effects, antagonistic weakening

## Abstract

The mixed application of plant residues to treat petroleum-contaminated soil may increase remediation efficiency by leveraging their mixed decomposition effects. However, it remains unclear whether this approach may also lead to opposite effects. In this study, plant residues of 7 common plant species, namely, *Bothriochloa ischaemum* (Bi), *Lespedeza davurica* (Ld), *Artemisia gmelinii* (Ag), *Heteropappus altaicus* (Ha), *Artemisia annua* (Aa), *Sophora davidii* (Sd), and *Agropyron cristatum* (Ac), from the petroleum-producing area of northern Shaanxi and their 9 mixtures were used to treat petroleum-contaminated soil with a crude oil content of 15.00 g kg^−1^. The samples were incubated at 25 °C under constant humidity for 120 days to determine the potential effects of mixed plant residues on stimulating contaminant degradation and restoring soil biological and chemical properties. The results revealed that (1) among all mixed plant residue treatments, combinations of BiLdAg, LdSd and HaAa significantly weakened the overall contaminant degradation efficiency. In particular, the LdSd and HaAa mixtures simultaneously strongly inhibited the degradation of saturated hydrocarbons and aromatic hydrocarbons; compared with the expected theoretical values, their degradation rates decreased by 12.36–35.25% and 9.91–19.82%, respectively (*p* < 0.05). (2) Mixtures including LdAgHa, BiLdAg, LdSd, LdAgHaAa, LdHa and HaAgAcSd significantly impaired the capacity of plant residues to replenish soil available nutrients and activate soil enzyme activities. The LdAgHa mixture exhibited the strongest antagonistic effect: its improvement efficiency in terms of soil nitrate nitrogen content and sucrase, dehydrogenase and polyphenol oxidase activities (the increase relative to that in contaminated soil) decreased by 30.60–76.89% relative to the theoretical expectations (*p* < 0.05). Overall, the effects of mixed plant residue addition on contaminant degradation and the restoration of damaged soil biochemical properties were negatively correlated. (3) Increased chemical specialization of mixed residues and higher mass proportions of Ld and Bi residues in mixtures tended to aggravate the above antagonistic inhibitory effects. When all the remediation indicators were comprehensively evaluated, the mixed application of plant residues failed to universally improve the remediation performance of crude oil-contaminated soil. Under experimental conditions, BiLdAg and LdSd residue combinations may lead to remarkable antagonistic interactions and lower the efficiency of necrophytoremediation, which should be avoided in practical field applications of this remediation technology.

## 1. Introduction

Petroleum is among the key raw materials and energy sources for modern industry. However, serious soil contamination often occurs because of leakage accidents during exploration, extraction, transportation, and refining processes. According to estimates from public data from the China National Petroleum Corporation, more than 8 million tons of crude oil enter the environment annually because of various accidents worldwide, a considerable portion of which enter the soil environment, causing direct and indirect economic losses of billions of dollars. In addition, the toxicity of petroleum contaminants and their long-term potential damage to soil physical, chemical, and biological properties, as well as the soil ecological functions controlled by these properties, are even more incalculable [1,2,3]. Therefore, efficient remediation of petroleum-contaminated soil has become one of the key scientific issues to be solved urgently in the fields of environmental science and soil science.

Necrophytoremediation is a bioremediation method that has received extensive attention in recent years [4,5]. It can significantly accelerate the degradation of contaminants and promote the recovery of damaged soil functions by providing shelter, nutrients, and easily available carbon sources and cometabolic substrates for microbial decomposers to degrade contaminants [4,5], thus promoting the restoration of soil ecological functions [6,7]. However, this technology still faces two major bottlenecks in practical applications. That is, some plant residues decompose quite slowly, making it difficult to effectively remove contaminants before they age [8]; in addition, given that plant residues usually have specific promoting effects on the degradation of different petroleum components, their ability to improve the physical, chemical, and biological properties of soils also differs [9]. The above problems restrict the application potential of this technology.

To overcome the limitations of necrophytoremediation using monospecific plant residue, many studies have explored improvement approaches using mixed plant residues or plant residues mixed with other organic residues to treat petroleum-contaminated soil and have achieved favorable results. For example, Ogbonna et al. [10] reported that the contaminant degradation efficiency of mixed treatment with cassava peels and sawdust on oil-contaminated soil was significantly greater than that of the corresponding single-species treatments. Udume et al. [11] reported that compared with monospecific residue treatments, mixed spent mushroom and water hyacinth composts had a significantly greater stimulating effect on the degradation of polycyclic aromatic hydrocarbons (PAHs) in unsterilized soil. Liu, Wang, Jing, Liang, Zhang, Liu and Zhang [9] reported that treatment with mixtures of green wastes from *Platycladus orientalis* and *Picea asperata* had significantly greater effects on contaminant removal and key enzyme activity improvement than expected. Recent studies have further revealed that one core mechanism underlying the aforementioned synergistic enhancement lies in the nonadditive effects generated during mixed decomposition. Such effects can accelerate the overall decomposition rate of plant residues, release abundant nutrients to stimulate decomposer growth within a short period, and simultaneously accelerate the release of degradation-promoting organic substances [12,13]. This process further optimizes the composition and functional structure of soil microbial communities [12,14], thereby improving the degradation efficiency of contaminants.

Nevertheless, the nonadditive effects of mixed decomposition are bidirectional [15]. Although numerous studies have verified their synergistic effects on remediation, a wealth of research has also indicated that inappropriate residue combinations may antagonistically suppress the overall decomposition of mixtures and impair their improvement effects on soil properties [12]. For instance, under uncontaminated conditions, the mixing of *Robinia pseudoacacia* litter with *Quercus liaotungensis* or *Caragana microphylla* litter significantly reduces their decomposition rates compared with theoretical expectations and simultaneously causes decreases in their stimulation or supplementation effects on soil dehydrogenase and sucrase activities, as well as soil organic matter and available nitrogen and phosphorus contents [12,16]. The above indicators play critical roles in the degradation of crude oil contaminants and directly govern the performance of soil ecological functions [17,18]. Research conducted by Liu et al. [9] further confirmed that certain mixed plant residue combinations, such as mixtures of *Platycladus orientalis* waste with *Acer truncatum* or *Pinus tabuliformis* waste, markedly weaken the removal efficiency of crude oil contaminants. In addition, the degradation of crude oil contaminants consumes massive amounts of soil nutrients [19], and the accumulation of highly toxic intermediate products inhibits soil enzyme activities [20]. Accordingly, inappropriate residue combinations may severely reduce the overall remediation capacity of plant residues; even when contaminant degradation is synergistically promoted, such combinations may have adverse effects on the recovery of soil biochemical properties and the soil ecological functions they represent. Given the wide range of plant residue sources and the limited yield of residues from a single species under practical application scenarios, the utilization of mixed plant residues for contaminated soil remediation is inevitable. Therefore, identifying and avoiding inappropriate residue combinations during technical development, as well as elucidating the underlying mechanisms driving antagonistic inhibitory effects, have important theoretical and practical significance. Furthermore, nonadditive effects arising from the mixed decomposition of plant residues are generally regulated by residue diversity (including species and chemical diversity) and the presence of specific plant residues (e.g., easily decomposable residues with high relative nutrient contents and low structural compound contents) [21]. Whether adjustments to these factors can alleviate the adverse effects of incompatible residue combinations on remediation performance remains to be verified.

The northern Loess Plateau constitutes one of China’s major oil-producing regions, yet it simultaneously suffers from severe soil contamination. Some previous local studies have focused on the potential of the mixed application of plant residues to synergistically remediate contaminated soil [22,23,24], but the potential risks posed by antagonistic effects to remediation efficiency have not been evaluated. To verify whether mixed plant residues lead to antagonistic effects on remediation performance and to preliminarily elucidate the underlying mechanisms and regulatory factors of such antagonism, this study selected residues of seven dominant local plant species, including *Bothriochloa ischaemum*. Mixtures were prepared on the basis of the primary composition patterns of natural plant communities in contaminated areas to remediate simulated crude oil-contaminated soil. This study aims to explore the potential negative effects of mixed residue application on the soil remediation performance of plant residues to provide scientific reference for the application and optimization of remediation technologies for crude oil-contaminated local soils.

Three hypotheses were proposed in this study: (1) the application of plant residues mixed in specific forms may cause antagonistic inhibition of the overall remediation efficiency for contaminated soil; (2) even if mixed plant residues increase the degradation of crude oil contaminants, they may simultaneously impose adverse impacts on the recovery of soil ecological functions; and (3) increased species (chemical) diversity of mixtures and the presence of easily decomposable plant residues may mitigate the magnitude of the aforementioned negative effects.

## 2. Results

**Figure 1:**
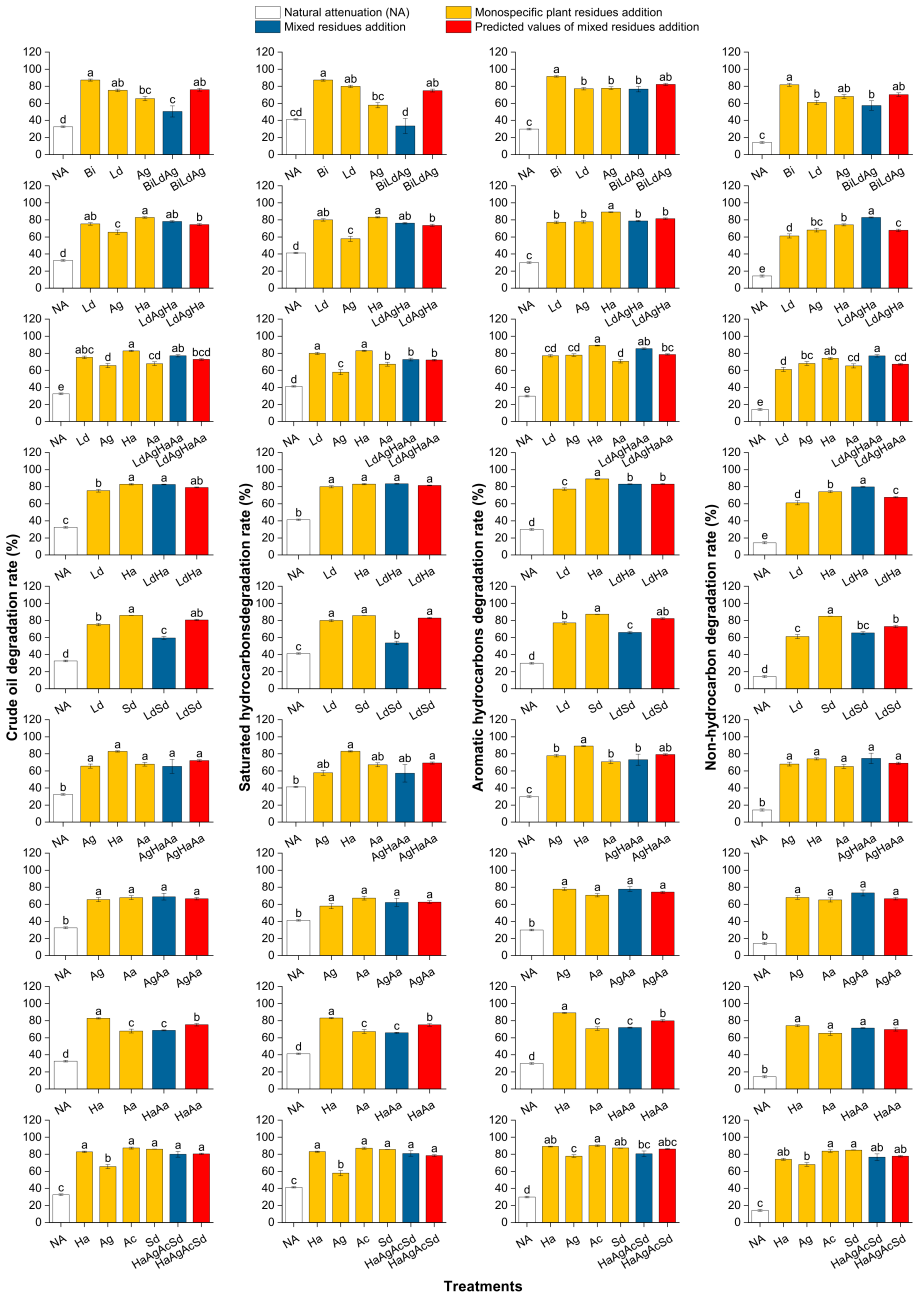
Degradation rates of crude oil and its components in contaminated soil after different plant residue treatments. Different letters within the subgraphs indicate significant differences among the various treatments (including the predicted values of mixed plant residue addition treatments) according to one-way analysis of variance (ANOVA), and Tukey’s HSD method was used for post hoc analysis, *p* < 0.05. When the measured values of specific mixed plant residue treatments are significantly lower than the theoretically predicted values, it indicates that mixing exerts antagonistic inhibition on the remediation effect of plant residues. Sd: *Sophora davidii*; Ag: *Artemisia gmelinii*; Aa: *Artemisia annua*; Bi: *Bothriochloa ischaemum*; Ld: *Lespedeza davurica*; Ha: *Heteropappus altaicus*; Ac: *Agropyron cristatum*.

Natural attenuation had a limited degradation effect on total petroleum contaminants (Figure 1). The degradation rates significantly increased after treatment with monospecific plant residues (*p* < 0.05). However, among the mixed plant residue treatments, mixed application of BiLdAg, LdSd, and HaAa plant residues significantly weakened the degradation effect of plant residues (*p* < 0.05).

When each component of contaminants was considered specifically, the degradation rates of saturated hydrocarbons, aromatic hydrocarbons, and nonhydrocarbon substances also significantly increased after treatment with monospecific plant residues (*p* < 0.05). Among the mixed plant residue treatments, mixed application of the aforementioned forms (especially LdSd and HaAa) also significantly weakened the degradation effects of plant residues on saturated and aromatic hydrocarbons (*p* < 0.05). However, it is worth noting that mixed application of LdAgHa, LdAgHaAa, and LdHa significantly promoted the degradation effect of plant residues on nonhydrocarbon substances (*p* < 0.05).

**Figure 2:**
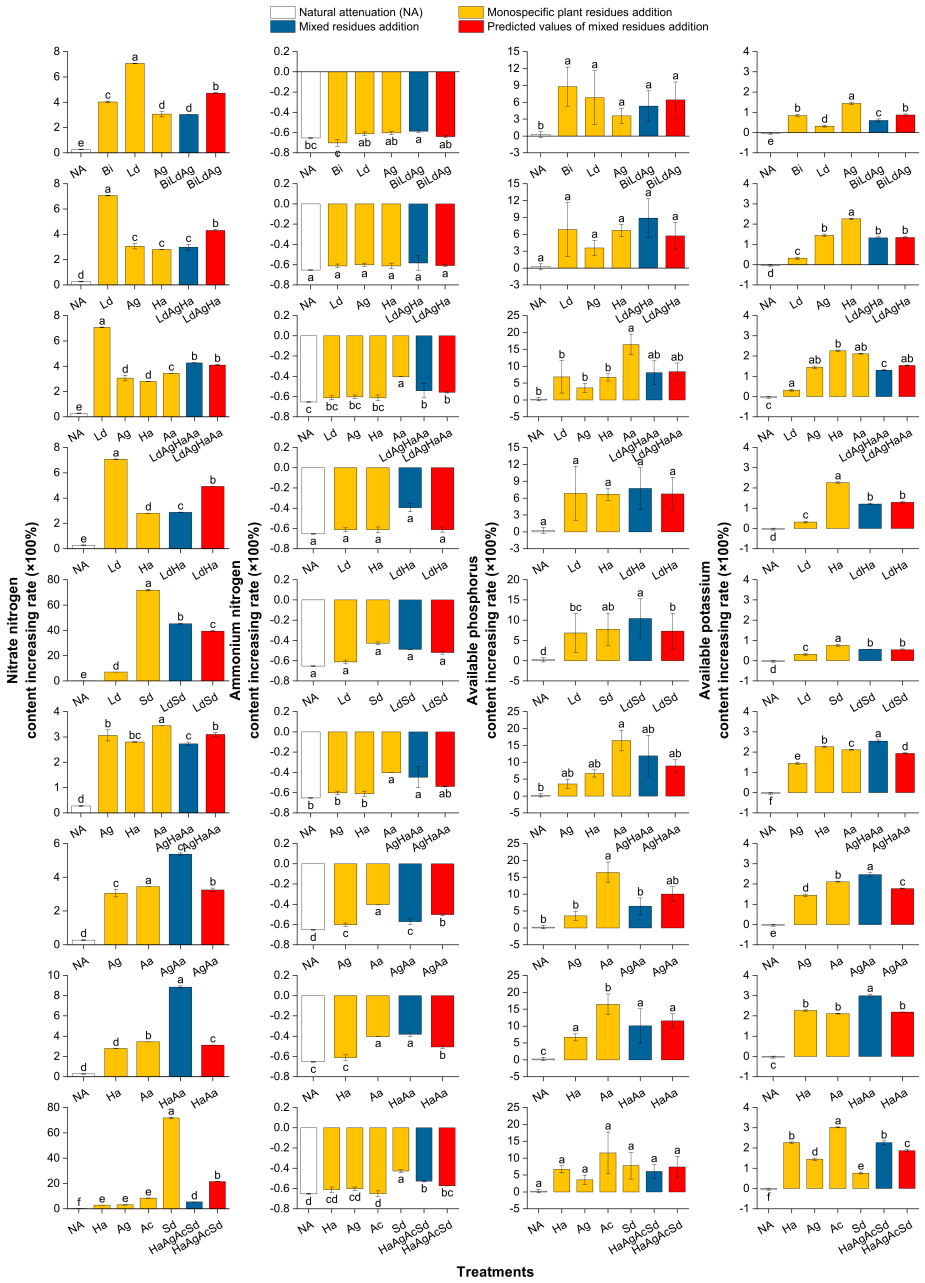
Soil available nutrient supplementation efficiency after different plant residue-treated contaminated soil. The supplementation efficiency of each treatment on soil available nutrients was characterized by an increase in the nutrient content relative to that of the contaminated soil. Different letters within each subplot denote significant differences among all treatments (including theoretically predicted values of mixed plant residue treatments) based on one-way analysis of variance (ANOVA), and Tukey’s HSD method was used for post hoc analysis, *p* < 0.05. When the measured values of specific mixed plant residue treatments are significantly lower than the theoretically predicted values, it indicates that mixing exerts antagonistic inhibition on the remediation effect of plant residues. Sd: *Sophora davidii*; Ag: *Artemisia gmelinii*; Aa: *Artemisia annua*; Bi: *Bothriochloa ischaemum*; Ld: *Lespedeza davurica*; Ha: *Heteropappus altaicus*; Ac: *Agropyron cristatum*.

Crude oil contamination significantly reduced the contents of soil available nitrogen and phosphorus (*p* < 0.05). Natural attenuation did not significantly influence these indicators (*p* > 0.05) and even caused further loss of ammonium nitrogen (*p* < 0.05; Table 1 and Figure 2). With the exception of a few individual cases, treatments with monospecific plant residues generally exhibited a significantly stronger capacity to replenish soil available nutrients (or mitigate the nutrient depletion induced by natural attenuation) than natural attenuation (*p* < 0.05; Figure 2), which reflected their supplementation effect on soil available nutrients.

However, in the mixed plant residue treatments, the mixed application of BiLdAg, LdAgHa, LdHa, AgHaAa and HaAgAcSd, AgAa, BiLdAg and LdAgHaAa significantly weakened the effects of plant residues on soil nitrate and ammonium nitrogen and available potassium supplementation (*p* < 0.05). However, mixed application of some forms (LdSd, AgAa, and HaAa, HaAa, and LdSd, as well as AgHaAa, AgAa, HaAa, and HaAgAcSd) significantly enhanced the effects of plant residues on the supplementation of soil nitrate and ammonium nitrogen, available phosphorus, and available potassium contents, respectively (*p* < 0.05; Figure 2).

**Figure 3:**
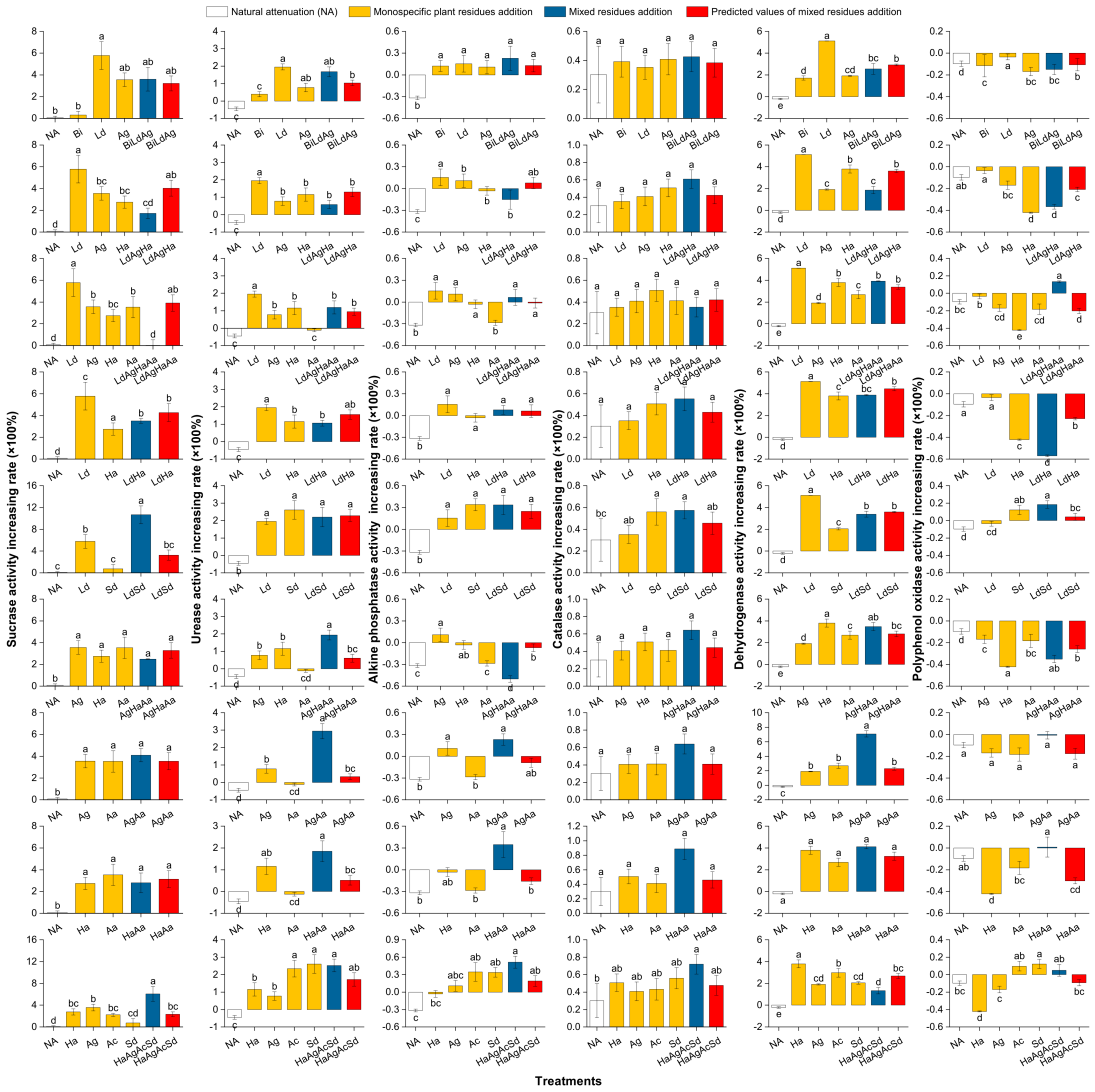
Soil enzymatic activity stimulatory effect after different plant residue-treated contaminated soil. The stimulatory effect of each treatment on soil enzyme activity was characterized by an increase in the rate of increase in enzyme activity relative to that of the contaminated control. Different letters within each subplot denote significant differences among all treatments (including theoretically predicted values of mixed plant residue treatments) based on one-way analysis of variance (ANOVA), and Tukey’s HSD method was used for post hoc analysis, *p* < 0.05. When the measured values of specific mixed plant residue treatments are significantly lower than the theoretically predicted values, it indicates that mixing exerts antagonistic inhibition on the remediation effect of plant residues. Sd: *Sophora davidii*; Ag: *Artemisia gmelinii*; Aa: *Artemisia annua*; Bi: *Bothriochloa ischaemum*; Ld: *Lespedeza davurica*; Ha: *Heteropappus altaicus*; Ac: *Agropyron cristatum*.

Crude oil contamination significantly inhibited soil urease activity but stimulated soil phosphatase activity. No significant increases in these enzyme activities were observed under natural attenuation; instead, the increase in enzyme activity induced by crude oil contamination decreased markedly (Table 2, *p* < 0.05). With the exception of catalase and polyphenol oxidase (the former was generally unaffected, while the latter was significantly suppressed in most cases), soil enzyme activities under the monospecific plant residue treatments were significantly greater than those under natural attenuation in most of the scenarios, indicating a stimulatory effect on enzyme activity (*p* < 0.05; Figure 3).

However, among the mixed plant residue treatments, mixed application in the forms of LdAgHa and LdAgHaAa, AgHaAa, LdAgHa and HaAgAcSd, as well as LdAgHa and LdHa, significantly weakened the effects of plant residues on stimulating soil sucrase, alkaline phosphatase, dehydrogenase, and polyphenol oxidase activities (*p* < 0.05). However, the combined application of LdSd and HaAgAcSd; AgHaAa; AgAa; HaAa; and HaAgAcSd; HaAa; HaAa and HaAgAcSd; AgAa and HaAa; and LdAgHaAa, AgAa, and LdSd significantly synergistically increased the effects of plant residues on the activity of sucrase, urease, phosphatase, catalase, dehydrogenase, and polyphenol oxidase, respectively (*p* < 0.05).

**Figure 4:**
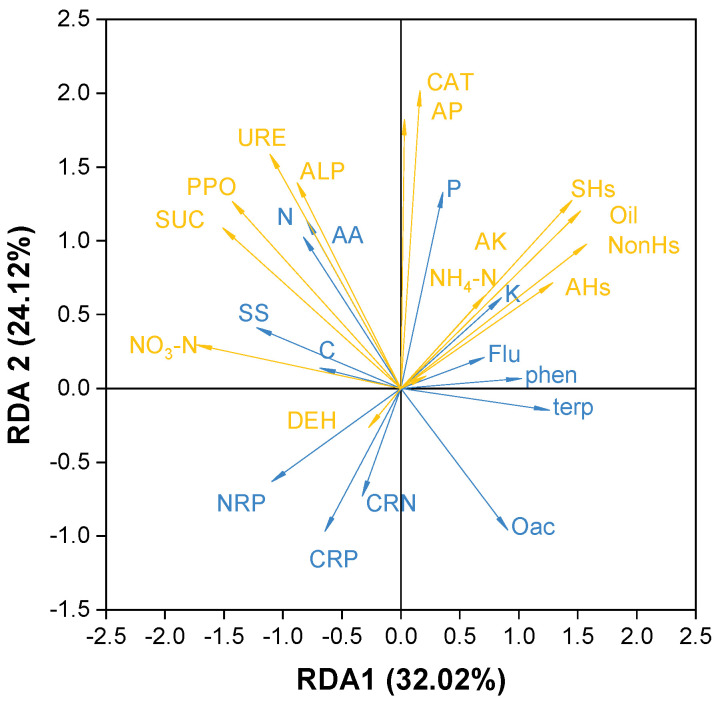
Chemical traits of plant residues (blue arrows, explanatory variable) and their remediating effects on crude oil-contaminated soil (yellow arrows, response variable) based on redundancy analysis. Oil, SHs, AHs and NonHs denote the degradation rates of total crude oil and its saturated hydrocarbons, aromatic hydrocarbons and nonhydrocarbon fractions, respectively. NO_3_-N, NH_4_-N, AP and AK represent the replenishment effects of mixed plant residues on soil nitrate nitrogen, ammonium nitrogen, available phosphorus and available potassium in contaminated soil, respectively; SUC, URE, ALP, CAT, DEH and PPO represent their stimulatory effects on the activities of sucrase, urease, alkaline phosphatase, catalase, dehydrogenase and polyphenol oxidase, respectively (which are calculated as the increase rate relative to the contaminated soil). C, N, P, K, phen, SS, AA, terp, Flu and Oac refer to the contents of carbon, nitrogen, phosphorus, potassium, polyphenols, soluble sugars, amino acids, terpenoids, flavonoids and organic acids in plant residues, respectively, whereas CNR, CPR and NPR denote the C/N, C/P and N/P ratios of plant residues, respectively.

Redundancy analysis (RDA) revealed that the chemical characteristics of mixed plant residues explained 56.14% of the total variation in remediation performance across all the treatments (Figure 4). Overall, the contents of amino acids, soluble sugars, total carbon and total nitrogen in the plant residues were mainly positively correlated with their capacity to replenish soil nitrate nitrogen and stimulate the activities of phosphatase, urease, sucrase and polyphenol oxidase. The contents of total phosphorus, total potassium, flavonoids, polyphenols and terpenoid secondary metabolites in the plant residues were positively correlated with their replenishment of soil ammonium nitrogen, available phosphorus and available potassium, together with the removal efficiency of total crude oil contaminants and their fractions. In contrast, the C/N, C/P and N/P ratios of the plant residues were negatively correlated with the above remediation indicators but positively correlated with the stimulatory effect on dehydrogenase activity.

**Figure 5:**
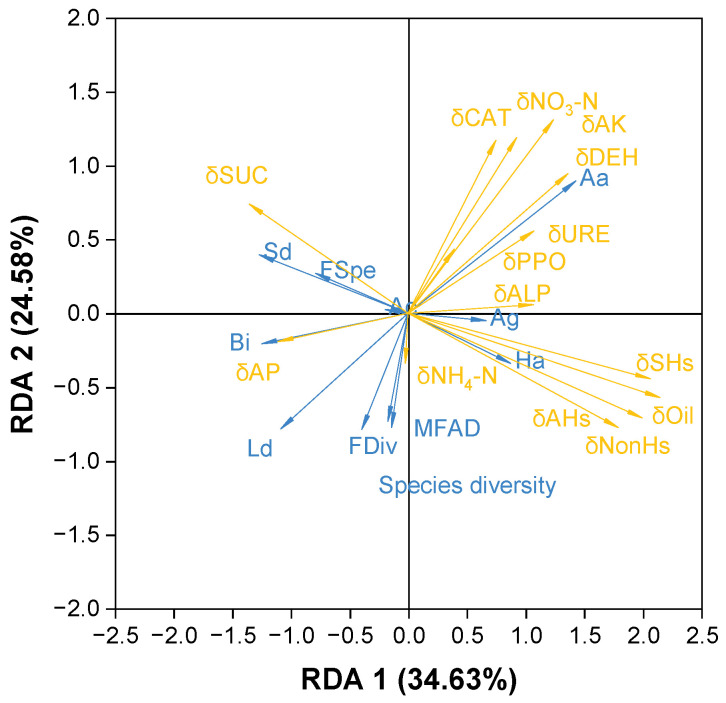
Relationships between the diversity and species composition of mixed plant residues (blue arrows, explanatory variable) and their ability to enhance soil remediation effects (that is, the increase rate (δ) of the measured values relative to the theoretically predicted values for the soil remediation performance of mixed plant residues, yellow arrows, response variable) based on redundancy analysis. Oil, SHs, AHs and NonHs denote the degradation rates of total crude oil and its saturated hydrocarbons, aromatic hydrocarbons and nonhydrocarbon fractions, respectively. NO_3_-N, NH_4_-N, AP and AK represent the replenishment effects of mixed plant residues on soil nitrate nitrogen, ammonium nitrogen, available phosphorus and available potassium in contaminated soil, respectively; SUC, URE, ALP, CAT, DEH and PPO represent their stimulatory effects on the activities of sucrase, urease, alkaline phosphatase, catalase, dehydrogenase and polyphenol oxidase, respectively (which are calculated as the increase rate relative to the contaminated soil). Sd: *Sophora davidii*; Ag: *Artemisia gmelinii*; Aa: *Artemisia annua*; Bi: *Bothriochloa ischaemum*; Ld: *Lespedeza davurica*; Ha: *Heteropappus altaicus*; Ac: *Agropyron cristatum*. MFAD, FDiv and FSpe represent the modified functional attribute diversity, functional divergence, and functional specialization indices, respectively, of mixed plant residues and were used to quantify the chemical diversity of mixed plant residues.

In terms of the enhancement mechanisms of remediation performance, the species diversity, chemical diversity and species composition of mixed plant residues collectively explained 59.21% of the total variation in the synergistic enhancing effects on remediation efficiency (Figure 5). Among these factors, residue diversity (including species diversity, chemical abundance and chemical dispersion), as well as the mass proportions of *Heteropappus altaicus* and *Artemisia gmelinii* residues, was positively correlated with the magnitude of increase in contaminant degradation and ammonium nitrogen replenishment effects (expressed as the rate of increase in the measured values relative to the theoretically predicted values). The chemical specialization of mixtures and the mass proportions of *Bothriochloa ischaemum*, *Lespedeza davurica* and *Sophora davidii* residues were negatively correlated with the above indicators but positively correlated with the magnitude of increase in sucrase activity stimulation and available phosphorus replenishment effects. The proportion of *Artemisia annua* residues was positively correlated with the magnitude of the stimulatory effects on dehydrogenase, urease, catalase, polyphenol oxidase and alkaline phosphatase activities, as well as the replenishment effects of nitrate nitrogen and available potassium. In contrast, the proportion of *Lespedeza davurica* residues was negatively correlated with all the aforementioned indicators. Furthermore, the two types of synergistic effects induced by mixed plant residue addition, i.e., the promotion of contaminant degradation and the restoration of damaged soil biochemical properties, were negatively correlated overall.

## 3. Discussion

### 3.1. Antagonistic Weakening of Contaminant Removal Efficiency Under Mixed Plant Residue Addition

Consistent with previous research results, the treatment of soil with plant residues can significantly stimulate the degradation of various contaminants. First, the nutrients released from plant residues may significantly stimulate the growth and degradation ability of petroleum-degrading microorganisms [12,25]. Moreover, plant residue-derived surface-active substances can promote the dissolution and dispersion of hydrophobic petroleum contaminants, and their cometabolic substrates might stimulate the secretion of microbial oxidoreductases [26,27,28]. The RDA results of this study (Figure 4) confirmed that the contents of amino acids and nitrogen and phosphorus in plant residues were positively correlated with their efficiency in replenishing available nutrients and stimulating the activities of dehydrogenase and catalase, and the latter indicators were positively correlated with the degradation of petroleum contaminants (especially saturated hydrocarbons and nonhydrocarbon substances). Moreover, the contents of phenolic and terpenoid secondary metabolites in plant residues were positively correlated with the degradation of each component of contaminants, which confirmed the stimulating effect of plant residue-derived nutrients and secondary metabolites on contaminant degradation.

However, consistent with hypothesis 1 of this study, inappropriate mixed applications of plant residues, such as BiLdAg, LdSd and HaAa, introduce significant antagonistic effects, leading to a significant reduction in contaminant degradation capacity compared with expectations (Figure 1). The reasons for the above inhibitory effects may include the following aspects. First, mixing different plant residues may negatively affect overall decomposition [29,30], markedly slowing the release rate of nutrients and contaminant-degrading stimulants from mixtures. Such inhibitory effects are generally more pronounced for high-quality plant residues with higher concentrations of nutrients and labile carbon sources [31]. For instance, our preliminary related investigation revealed that under uncontaminated conditions (sharing the same source of soil and sampling and processing method as the present study, the same hereafter), the total mass loss of mixed Bi, Ag and Ld residues, which was only stub-cut but not pulverized, after 180 days of decomposition was approximately 13% lower than the theoretically predicted value. The decomposition rate of nutrient-rich Ld residues decreased by nearly 20% (unpublished data). As shown in Table S1, the nitrogen and phosphorus contents of the Ld residues were 9.03–92.97% and 2.75–5.53 times greater than those of the other two residues, respectively (*p* < 0.05). In contrast, excessive nitrogen supplied by high-quality plant residues may restrict the synthesis and release of phenolic compounds linked to lignin degradation within mixtures [32]. In the present study, the release of phenolic compounds such as polyphenols and flavonoids from Ag residues may have been subject to similar restrictions. Consequently, even if all the nutrients and degradation stimulants are fully released by the end of incubation, optimal remediation performance cannot be achieved before contaminant aging occurs. Second, previous research has confirmed that mixing distinct plant residues can provide decomposers with more balanced and diversified substrates. In addition, the material transfer between different plant residues greatly improves their decomposability, leading to decomposition rates significantly higher than predicted values [33,34]. However, the rapid decomposition of mixed residues may shift soil microorganisms toward preferentially utilizing plant substrates whose stoichiometric ratios match their own metabolic demands rather than crude oil carbon sources characterized by severe N and P deficiency and strong biological toxicity. The microbial community structure and functional profiles shaped by mixed plant residue decomposition may therefore become less suitable for contaminant degradation, resulting in weaker remediation efficiency relative to theoretical expectations. For example, under uncontaminated conditions, the total mass loss of mixed Ld and Sd residues after 180 days of decomposition was 12.57% greater than predicted. Nevertheless, when applied to crude oil-contaminated soil, which was totally the same as that used in this study (the same as hereafter), the relative abundances of soil bacterial functional pathways directly involved in crude oil degradation, including hydrocarbon degradation, aliphatic nonmethane hydrocarbon degradation and aromatic hydrocarbon degradation, decreased significantly. Moreover, pathways associated with plant residue decomposition, such as fermentation (degradation of simple sugars and hemicellulose) and ligninolysis (lignin degradation), were markedly enriched (unpublished data). This represents one critical mechanism explaining why the LdSd combination reduces the contaminant removal efficiency of plant residue treatments. In addition, plant residues generally contain insufficient amounts of nitrogen and phosphorus to sustain their decomposition processes. Decomposers therefore actively immobilize available nutrients from the surrounding soil during plant residue decomposition [35,36,37]. Further depletion of soil-available N and P strongly inhibited crude oil degradation. Finally, the accelerated decomposition capacity of decomposers induced by mixed plant residue decomposition may lead to complete mineralization of secondary metabolites, which is beneficial for contaminant degradation during plant residue decomposition. For example, Li et al. [13] reported that most plant residue mixtures composed of *P. massoniana*, *Sassafras tzumu*, *Cinnamomum camphora* and *Toona sinensis* exhibited significantly higher degradation rates of total phenols and condensed tannins than predicted values during mixed decomposition. This phenomenon may drastically weaken the capacity of mixed plant residues to stimulate contaminant degradation.

Notably, three types of plant residue mixtures (LdAgHa, LdAgHaAa and LdHa) significantly increased the removal efficiency of nonhydrocarbon fractions, whereas no other mixed form exhibited any antagonistic effects. This discrepancy can be attributed to the intrinsic properties of nonhydrocarbon substances (asphaltenes and resins), which contain abundant polar functional groups and bind tightly to soil particles, resulting in a far lower bioavailability than that of saturated and aromatic hydrocarbons [38,39]. Accordingly, nonhydrocarbon fractions are less sensitive to shifts in soil nutrient status and soil microbial functions caused by the addition of mixed plant residue. The synergistic promotion of the synthesis and release of surface-active components (organic acids and humic acids) driven by mixed plant residue application is highly likely the primary mechanism through which these three mixtures improve nonhydrocarbon removal efficiency [39]. Nevertheless, the present study and our previous experiments did not quantify the effects of these mixtures on the release of organic acids or other surface-active substances or on the humification processes of plant residues. Further experimental verification is needed to validate the above speculation in follow-up research.

### 3.2. Antagonistic Weakening of Damaged Soil Biochemical Properties—Improving Efficiency Under Mixed Plant Residue Addition

Similar to previous studies, plant residue treatment can significantly increase soil available nutrient contents and enzyme activities. The reason is that the nitrogen, phosphorus, and potassium released by plant residues directly supplement the available nutrient contents in the soil and stimulate the secretion of nutrient transformation-related enzymes [40,41]. In addition, studies have shown that the functions of nitrogen-fixing/transforming, phosphorus- and potassium-solubilizing microorganisms in soil (including the synthesis and secretion of related enzymes) are restored after plant residue treatment [24,42,43], which also promotes the transformation of total soil nutrients into available forms.

However, consistent with our initial hypothesis, the partial mixed plant residue treatments also significantly reduced the nutrient replenishment capacity and enzyme-stimulating effects (Figure 2 and Figure 3). The underlying mechanisms have been described above. On the one hand, mixing plant residues exerts antagonistic inhibition on their decomposition [33,44]. This slows the breakdown of plant residue tissue structures; organic matter such as proteins and phospholipids cannot be rapidly hydrolyzed or mineralized to release nutrients, and microbial enzyme synthesis and secretion are constrained by insufficient substrate supply. As mentioned previously, the BiLdAg mixture significantly suppressed overall decomposition, especially for nutrient-rich Ld residues (unpublished data and Table S1), which in turn slowed nutrient release. The limited nutrients released gradually are continuously consumed during contaminant degradation [45], ultimately lowering the final nutrient replenishment efficiency below theoretical predictions.

All the plant residues were fully decomposed by the end of the incubation period in this study; thus, variations in the decomposition rate did not alter the total amount of nutrients released. Accordingly, we infer that the antagonistic reduction in soil nutrient replenishment observed for most mixtures stems mainly from the synergistic promotion of plant residue decomposition or contaminant degradation induced by mixing. The application of mixed plant residue drastically increased the soil microbial biomass. Given the fixed stoichiometric demands of microorganisms for carbon, nitrogen and phosphorus during cell construction, protein synthesis, energy metabolism and genetic replication, decomposers immobilize scarce N and P from the soil to sustain their growth and metabolism [45]. Although microbial indicators were not measured in this study, relevant literature has reported that even low-quality plant residues such as *P. tabuliformis*, *Larix gmelinii* and *Platycladus orientalis* synergistically increase the populations of total culturable soil bacteria and fungi when mixed with high-quality *R. pseudoacacia* litter, with microbial counts rising 60% to more than 9.5 times higher than the predicted values [12]. This synergistic stimulation persists even when plant residue decomposition itself is unaffected or antagonistically suppressed [16]. Such massive microbial proliferation inevitably depletes soil nutrients, resulting in an apparent antagonistic weakening of the nutrient supplementation effect of plant residues.

Moreover, enhanced contaminant degradation leads to the accumulation of toxic intermediate metabolites, which ultimately inhibit nutrient transformation functions of soil microorganisms through feedback [46]. Consequently, soil available nutrient concentrations and enzyme activities decrease significantly below predicted levels. This finding serves as the primary explanation for the antagonistic suppression of soil nutrient restoration and enzyme activity recovery observed in mixtures including LdAgHa, LdAgHaAa and LdHa, which exhibited a trending or significant synergistic promotion of degradation for certain contaminant fractions (Figure 1). The redundancy analysis results of this study verified the rationality of the above inference: the nutrient replenishment and enzyme-stimulating effects of mixed plant residue treatments (Figure 4), as well as the enhancement magnitudes of these remediation functions (Figure 5), were nearly all negatively correlated with indicators of contaminant degradation.

### 3.3. Relationships Between Plant Residue Diversity, Species Composition and Nonadditive Effects Induced by Mixed Residue Addition

Redundancy analysis (Figure 5) revealed that increased species diversity, chemical diversity and divergence, as well as increased proportions of Ha and Ag residues, tended to significantly promote the degradation of contaminants in soil when mixed plant residues were applied. In contrast, increased chemical specialization and increased proportions of Bi and Sd residues tended to produce antagonistic effects.

This finding first indicates that when the number of plant residue species is low and the chemical properties of different plant residues are similar, the degree to which mixed application weakens their remediation effects is more obvious. The reason may be that in the above cases, the complementarity and comprehensiveness of nutrients provided by different plant residues to their own decomposers and contaminant decomposers are lower. This leads to the fact that the mixed addition of different plant residues not only fails to stimulate the growth and activity of the two types of microorganisms but also may even induce competition between different microbial groups that utilize similar substrates [47], thereby limiting the overall decomposition and nutrient release rate of plant residues and ultimately weakening the ability of microorganisms to degrade contaminants.

In addition, in contrast to previous findings, an increased proportion of Sd residues with optimal nutrient status reduced the contaminant degradation capacity of the residue mixtures. A possible explanation is that crude oil contamination drastically increases the soil C/N ratio [48]. Sd residues have extremely low C/N and N/P ratios and structural compound contents (Table S1); thus, they supply abundant nutrients to increase microbial proliferation. Moreover, massive Sd input preferentially stimulates the growth of microorganisms that rely on labile carbon substrates in soil [49], reshaping community structure and functional traits and consequently suppressing the microbial utilization of contaminant substrates. The presence and increasing proportion of Bi residues with similar nutrient statuses (Table S1) may attenuate the remediation performance of residue mixtures via a comparable pathway. Our unpublished data also revealed that treatments containing Sd or Bi residues resulted in significantly greater functional abundances of fungal litter saprotrophs (degrading simple organic compounds in plant residues) and bacterial fermentation pathways than hydrocarbon degradation pathways did, which supports the above inference.

In contrast, a higher proportion of Ha and Ag residues facilitated synergistic promotion of contaminant degradation in mixed residue treatments. As discussed earlier, this can be attributed to their high concentrations of phenolics, terpenoids and organic acids (Table S1). Organic acids may increase contaminant desorption and microbial uptake [50], whereas phenolic and terpenoid compounds might act as cometabolic substrates to stimulate the enzymatic degradation of contaminants [27]. In terms of soil microbial composition, the relative abundance of the typical oil-degrading bacteria *Nocardioides* increased significantly by 19.15% when Ag residues were present (unpublished data). Notably, the functional role of Ag residues was inconsistent across our series of parallel studies [22,51], which may be driven by variable residue species combinations and complex interactions among residues governing decomposition and remediation efficiency. The detailed underlying mechanisms require further validation.

Finally, consistent with our Hypothesis 3 and previous research, mixtures that weakened the contaminant degradation capacity frequently had stronger effects on replenishing soil available nutrients and stimulating soil enzyme activities [51]. This pattern may have occurred because plant residue combinations exerting antagonistic effects on contaminant degradation resulted in markedly slower decomposition rates. Nutrients released from such residues were less readily immobilized and consumed by microbes for contaminant breakdown, leading to an apparent significant increase in soil available nutrient concentrations. Furthermore, large quantities of organic matter released during plant residue decomposition serve as substrates to induce the secretion of diverse extracellular enzymes, driving a similar upward trend in soil enzyme activity. Nevertheless, nutrient translocation in this study was confined by the microcosms, while soils contaminated under natural conditions display strong hydrophobicity. We cannot rule out the possibility that nutrients returned to soil via plant residue decomposition become more susceptible to leaching loss, which may cause further latent damage to the compromised ecosystem function of oil-contaminated soil layers.

### 3.4. Issues to Be Clarified and Limitations of the Present Study

First, when the effects of mixed plant residue addition on remediation performance were evaluated, this study focused on the removal of crude oil contaminants and the restoration of impaired soil ecological functions. The soil ecological functions evaluated included four main aspects: (1) maintaining biogeochemical cycles, (2) supporting vegetation growth, (3) providing habitats for soil organisms, and (4) buffering and purifying environmental contamination. In terms of selected indicators, the degradation rates of total crude oil and its fractions mainly reflect functions 1–3; soil available nutrient concentrations primarily represent functions 2 and 3; and key soil enzyme activities reflect functions 1 and 4. Crude oil pollution generally depletes soil available nutrients intensely, inhibits the transformation of total nutrients into available forms, and suppresses soil enzyme activities via physiological toxicity and physical–chemical disturbance. Therefore, in this study, the rates of increase in contaminant degradation rates and soil biochemical indicators relative to those of contaminated soil (controls) were used to characterize the degree of remediation and ecological function restoration capacity of each treatment.

It should be noted, however, that this work measured only the extent of degradation of crude oil contaminants and the recovery or elevation of nine soil biochemical indicators to reflect the potential of plant residues to restore partial soil functions. The full expression of soil ecological functions relies on the synergistic action of multiple soil properties, and the recovery trajectories of different indicators are often decoupled. For example, the results in Figure 5 indicate that the elimination of soil crude oil contaminants may occur at the cost of accelerated nutrient consumption. Accordingly, a single category or individual indicator cannot quantify the complete recovery of soil ecological functions, and the minimum recovery thresholds of relevant indicators to reflect such trade-offs remain undetermined. Future research should integrate measurements covering local plant seed germination, vegetative growth, reproductive growth and community stability, together with the recovery of soil microbial community structure and functions, to comprehensively assess how residue mixing alters remediation efficiency.

Second, mixing modulates only the decomposition rates rather than the total amount of elements or specific organic compounds released into the soil, and all the tested plant residues achieved complete decomposition under the experimental conditions. The decomposition rate, mass loss, release of organic components, and nutrient immobilization–mineralization dynamics of mixed residues under oil-contaminated conditions were not measured in this study. The interpretation of the current results relies solely on previous decomposition data of intact or naturally fragmented litter under uncontaminated conditions, as well as nonadditive effects of mixing on litter decomposition rates. Although previous studies have indicated that fragmentation of plant residues does not hinder their non-additive effects on their own decomposition and soil properties [12], the soil media characteristics and sizes of plant residues in those aforementioned studies are not fully consistent with those in the present study. Thus, it cannot be ruled out that the effects of mixed decomposition on nutrient dynamics and the degradation and release of organic matter associated with contaminant degradation may differ from those reported in our earlier unpublished studies after plant residues are fragmented. Future research should directly examine the impacts of residue mixing on plant residue decomposition as well as the release and transformation of substances under conditions with contaminants and highly fragmented plant residues, so as to further elucidate the mechanisms underlying the findings of this study.

Third, to achieve timely remediation of crude oil contamination and eliminate uncertainties in the duration of contamination and degree of aging associated with randomly sampled field-contaminated soils, artificially freshly contaminated soil was used as the test substrate. This setup complies with the principle of immediate treatment upon contamination and minimizes biases induced by inconsistent degrees of contaminant aging. Nevertheless, before widespread effective environmental supervision occurred, many contaminated sites failed to receive timely remediation, and aged oil contaminants were widespread across the study area. Existing studies demonstrate that contaminant mobility and bioavailability strongly influence remediation performance in aged contaminated soils. Thus, mixing-induced shifts in the release of potential surfactant-like organic compounds from plant residues may drastically influence remediation efficiency. Extended experiments covering gradients of degrees of contaminant aging are needed in subsequent research to verify whether and to what extent aging modulates the remediation capacity of mixed plant residues to improve the generalizability of the findings in this study.

Finally, it is undeniable that plant residues inevitably carry a certain number of phyllosphere microorganisms into the soil, and the input of these exogenous microorganisms may exert a certain bioaugmentation effect [52]. However, the plant residues used in this study were newly formed standing dead residues and freshly fallen leaves. The phyllosphere microorganisms attached to these residues are predominantly composed of pathogens and saprophytic groups involved in the initial stage of plant residue decomposition (and microbial succession may not even have reached this stage) [53,54], which are vastly different from the microbial communities capable of degrading complex petroleum components, such as long-chain saturated hydrocarbons, aromatic hydrocarbons, and other non-hydrocarbon organic compounds. In addition, no strict aseptic treatment was performed throughout the experiment, which is consistent with practical field application scenarios. Moreover, the plant residues were subjected to relatively high-temperature pretreatment during the experiment, which may have introduced exogenous miscellaneous microorganisms and inactivated part of the indigenous phyllosphere microbes. Therefore, the interference of plant residues on contaminant degradation and soil properties via the bioaugmentation pathway, as well as the potential effects of mixed residue application on the input of degrading microorganisms, can be neglected in this study. Furthermore, due to experimental limitations, the present study explains the effects of mixed plant residue application on soil contaminant degradation and soil biochemical properties merely based on a small number of unpublished preliminary data from us. Although these preliminary data share consistent material sources and basic physicochemical characteristics with the current study, discrepancies exist in residue combination patterns and experimental duration. Accordingly, further analysis of the microbiological mechanisms underlying the experimental results requires subsequent detection of the microbial community structure and functional characteristics of the soil samples collected in this study.

## 4. Materials and Methods

### 4.1. Sampling Region

The test soil, plant residues and crude oil were all collected from the Yanjiawan oil production zone in Yanchang County, Yan’an City (109.9667° E, 36.5833° N, altitude 1026 m a.s.l.). The local area belongs to the Loess Hilly Region, which has a warm temperate continental semiarid monsoon climate. The annual average temperature is 10.4 °C, the annual average precipitation is 564 mm, and the annual sunshine hours are 2505 h.

The vegetation type corresponds to a transition zone between deciduous broad-leaved forest and steppe. The secondary shrub-grassland is dominated by monodominant and codominant communities composed of *Sophora davidii* (Sd), Artemisia species, including *Artemisia gmelinii* (Ag) and *Artemisia annua* (Aa), *Bothriochloa ischaemum* (Bi), *Lespedeza davurica* (Ld), *Heteropappus altaicus* (Ha), and *Agropyron cristatum* (Ac). The major community assemblages include BiLdAg, LdAgHa, LdAgHaAa, LdHa, LdSd, AgHaAa, AgAa, HaAa, and HaAgAcSd. The primary soil type is calcic cambisol (FAO classification), with a bulk density of approximately 1.1 g cm^−3^, a total porosity ranging from 50% to 65%, a soil organic matter content of approximately 0.55%, and a pH value between 8 and 8.5. The oil wells in the sampling area feature low single-well yields and scattered distributions. Difficult daily inspection together with aging pipeline facilities frequently causes minor drips during oil extraction and leakage induced by pipeline corrosion perforation, resulting in irregular patchy and spot oil contamination with uneven contaminant concentrations across local soils. Wide-range random sampling was conducted within the contaminated zones, and the gravimetric method with dichloromethane extraction was adopted for measurement. The total crude oil concentration in soils surrounding leaky wells and pipelines ranged from 11.44 to 44.72 g kg^−1^. Unless otherwise specified, the term “contaminants” refers to total crude oil, encompassing its saturated hydrocarbon, aromatic hydrocarbon, and all nonhydrocarbon fractions [23,24].

### 4.2. Simulated Remediation Experiment

#### 4.2.1. Collection and Pretreatment of Test Materials

As mentioned above, crude oil contamination in the study area is heterogeneously distributed, and in situ simulated remediation trials have limited treatment ranges. In field trials, it is difficult to control and quantify contaminant concentrations and the actual degree of aging in treated plots. Moreover, target indicators, including contaminant degradation rates, topsoil available nutrient contents and enzyme activities, are strongly disturbed by exogenous plants and their residue inputs, as well as soil wind and water erosion. Therefore, in this study, uncontaminated local soil was collected to artificially prepare oil-contaminated soil, and simulated remediation was carried out under controlled laboratory conditions. The experiment simulated a scenario in which necrophytoremediation is implemented shortly after crude oil leakage during extraction and transportation.

For soil sampling and pretreatment, ten 1 m × 1 m quadrats were randomly established on slopes around oil wells in the study area. All topsoil at 0–10 cm depth was collected, fully homogenized and sieved through a 1 mm mesh to remove plant and animal debris. Subsampling was conducted via the quartering method, and sufficient soil was transported back to the laboratory. Dichloromethane extraction–gravimetry confirmed that no crude oil components were present in the soil, and water immersion verified the absence of plant residue fragments.

Test crude oil was purchased from local oil wells, with a freezing point of 19.6 °C and a density of 0.881 g cm^−3^ (20 °C). The mass fractions of saturated hydrocarbons, aromatic hydrocarbons and nonhydrocarbon substances (asphaltenes, waxes, etc.) were 51%, 28% and 21%, respectively.

In autumn of the same year of soil sampling, dead aboveground standing litter and fallen leaves of seven dominant native species (*B. ischaemum*, *L. davurica*, *A. gmelinii*, *H. altaicus*, *A. annua*, *S. davidii*, and *A. cristatum*) were collected from uncontaminated slopes near oil wells. Standing litter was harvested 1 cm above ground; partially decomposed and insect-damaged fractions were discarded before homogenization within each species. For *Sophora davidii*, only natural fallen leaves were collected using ten 1 m × 1 m litter traps placed under the shrubs. All the plant residues were oven-dried at 65 °C and cut into fragments of approximately 2 mm for subsequent use. Previous studies have shown that this cutting treatment accelerates plant residue decomposition to shorten the incubation time without altering the nonadditive effects of mixed plant residue decomposition or their nonadditive effects on soil properties [12].

#### 4.2.2. Implementation of the Simulated Remediation Experiment

The saturated water capacity and initial moisture content of the test soil were measured first. A portion of the soil was air-dried and stored in a cool, ventilated place as the uncontaminated control (Unc, n = 3).

The remaining soil mass was converted to oven-dried mass for artificial contamination and remediation treatments; all mass values hereinafter refer to the oven-dried mass. On the basis of the widely distributed low contamination level in the field (11.44 g kg^−1^), the simulated contamination concentration was set to 15.00 g kg^−1^. To prepare the contaminated soil, 10 kg of oven-dried soil was weighed, part of which was thoroughly kneaded manually with 150 g of crude oil. The oil-soaked soil was then blended with the remaining clean soil, sieved repeatedly through a 1 mm mesh for homogenization, and placed in the shade for 15 days. This procedure enables the full dispersion and homogenization of crude oil without significant natural attenuation but causes remarkable deterioration of soil biochemical properties, which is suitable for evaluating plant residue remediation efficiency. A subsample of artificially contaminated soil was air-dried and stored as the light oil-contaminated control (LC, n = 3).

On the basis of the species compositions of the codominant plant communities described in Section 4.1, nine plant residue mixtures matching field community assemblages were prepared. Referring to the actual biomass proportions of each species in natural communities and to eliminate confounding effects from variable mixing ratios, equal mass proportions were adopted for all the species in each mixture. Monospecific or mixed plant residues were incorporated into 200 g of contaminated soil at a residue-to-soil ratio of 2% (*w*/*w*). Distilled water was added to adjust the soil moisture to 50% of the saturated water capacity. Each mixture was transferred into a custom-made incubation microcosm (22 cm × 16.3 cm × 9 cm) lined with polytetrafluoroethylene film. The breathable lid was sealed, and the total weight of each microcosm was recorded before incubation at a constant temperature of 25 °C for 120 consecutive days.

Every two weeks, the microcosms were weighed, and distilled water was evenly supplemented by opening the lid to replenish water loss and maintain stable soil moisture. A contaminated soil treatment without plant residue addition was considered natural attenuation (NA, control/treatment). All the incubation treatments were established in triplicate (Table 3).

After the incubation was complete, destructive sampling was performed for all microcosms. A portion of the soil samples was soaked in water; no intact floating plant residue fragments were observed, confirming the complete decomposition of the test plant residues before subsequent index determination.

### 4.3. Determination of Soil and Plant Residue Properties

The total contaminant content of the soil was determined by the dichloromethane extraction–gravimetric method. The extracted contaminants were subsequently redissolved and transferred to an activated silica gel-alumina chromatography column. Saturated hydrocarbons, aromatic hydrocarbons, and nonhydrocarbon substances were eluted and separated using n-hexane, a dichloromethane-n-hexane (2:1) mixture, and methanol, respectively. Their masses were determined, and the degradation rates were calculated.

The activities of soil polyphenol oxidase, catalase, dehydrogenase, urease, alkaline phosphatase, and sucrase were determined by the pyrogallol colorimetric method, potassium permanganate titration method, triphenyltetrazolium chloride colorimetric method, indophenol blue colorimetric method, disodium phenyl phosphate colorimetric method, and 3,5-dinitrosalicylic acid colorimetric method, respectively [55]. The contents of soil nitrate nitrogen, ammonium nitrogen, available phosphorus, and available potassium were determined by potassium chloride extraction–indophenol blue colorimetric method–ultraviolet spectrophotometry, phosphomolybdenum blue colorimetric method, flame photometry, and glass electrode method (soil–water ratio 2.5:1), respectively [56].

For the reserved undecomposed plant residue samples, the organic carbon content was determined by the concentrated H_2_SO_4_-K_2_Cr_2_O_7_ oxidation-FeSO_4_ titration method; their total nitrogen, total phosphorus, and total potassium contents were determined by the Kjeldahl method, vanadium molybdenum yellow colorimetric method and flame photometry, respectively, after the plant residues were digested with concentrated H_2_SO_4_-30% H_2_O_2_ [56]. The contents of soluble sugars, amino acids, total organic acids, water-soluble total phenols, terpenoids, and flavonoids were determined by the anthrone colorimetric method, ninhydrin-ethanol colorimetric method, potentiometric titration method, Folin-phenol colorimetric method, vanillin-glacial acetic acid colorimetric method, and sodium nitrite–aluminum nitrate method, respectively. The results are represented by the contents of monomers such as glucose, leucine, oxalic acid, gallic acid, ursolic acid, and rutin [57,58,59,60,61,62].

The detailed measurement procedures were identical to those described previously [55,56,57,58,59,60,61,62]. Only the testing instruments were updated: a K-375 automatic Kjeldahl distillation unit (Buchi, Flawil, Switzerland, for Kjeldahl nitrogen determination), a Titertte digital bottle-top burette (Brand, Wertheim, Germany, for titration analysis), an EPOCH 2 fully automated microplate reader (BioTek, Winooski, VT, USA, for visible light colorimetry), and a UV-2600 ultraviolet spectrophotometer (Shimadzu, Kyoto, Japan, for UV colorimetry). All analytical-grade reagents were purchased from Sinopharm Chemical Reagent Co., Ltd. (Shanghai, China) or Solarbio Science & Technology Co., Ltd. (Beijing, China).

### 4.4. Data Processing and Statistical Analysis

The remediation efficiency of each treatment for contaminated soil and the restoration of ecological functions were characterized by the degradation rates of total crude oil contaminants (and their individual fractions) and the increase rates of soil biochemical indicators relative to those of the contaminated soil, denoted as *RE_i_* (dimensionless decimal, Equation (1)). *RE_i_* was further divided into contaminant removal efficiency, soil available nutrient replenishment efficiency, and enzyme activity stimulation efficiency [17,63].

*RE_i_* = (*I_treat_* − *I_LC_*)/*I_LC_* (1)

where *I_treat_* and *I_LC_* represent the measured value of a given indicator under each type of plant residue addition treatment and that of the corresponding indicator determined in contaminated soil, respectively. Positive values indicate that soil biochemical indicators after treatment exceed those in the contaminated soil, indicating recovery of impaired indicators and ecological functions; negative values indicate deterioration of the indicator and its associated functions.

Equation (2) was subsequently adopted to calculate the theoretically predicted values (*Pre_ij_*) of the above indicators for each mixed plant residue treatment on the basis of the data from the monospecific treatments. Equation (3) was then used to quantify the magnitude of alteration induced by plant residue mixing (*δ_ij_*) on their remediation effects:

*Pre_i,j_* = Σ*S_i_* × *R_i_* (2)

*δ_i,j_* = (*Obs_i,j_* − *Pre_i,j_*)/*Pre_i,j_* (3)

where *S_i_* is the value of soil indicators after remediation using a given monospecific plant residue, *R_i_* is the mass proportion of corresponding plant residues in the combination, and *Obs_ij_* is the value of soil indicators after remediation using a corresponding mixed plant residue.

SPSS 23.0 software was used for one-way analysis of variance (ANOVA) to detect differences in chemical indicators among plant residues and to detect significant differences in the measured values of bio and chemical indicators of uncontaminated soil, contaminated soil, natural attenuation-treated soil, and soil samples treated with combinations of plant residue (predicted and observed values, *Pre_ij_* and *Obs_ij_*) and their corresponding monospecific plant residue types. When *Obs_ij_* < *Pre_ij_* < *S_ij_*… or *S_i_* < *Obs_ij_* < *Pre_ij_* < *S_j_* and the difference is significant, it can be considered that mixed treatment of oil-contaminated soil with plant residues can significantly weaken the remediation effect of each other on specific soil properties; otherwise, it indicates a synergistic enhancement effect. Tukey’s honestly significant difference (HSD) method was used for post hoc comparisons, and the significance test level was α = 0.05.

FDiversity software (http://www.fdiversity.nucleodiversus.org/) was used to calculate the community weighted mean (CWM traits) and chemical diversity indicators (including modified functional attribute diversity, MFAD, functional divergence, FDiv, and functional specialization, FSpe) of each chemical indicator of mixed plant residues on the basis of the chemical properties of the corresponding plant residues.

OriginPro 21.0 software was used for redundancy analysis (RDA) to detect the relationships between the overall chemical characteristics of mixed plant residues (including the CWM of nutrient contents and stoichiometric ratios) and their remediation effect indicators (degradation rates of total contaminants and their components, as well as soil bio and chemical indicators) and between the species and chemical diversity of mixed plant residues and their corresponding *δ_ij_* values.

All the graphs were constructed using OriginPro 2021b software.

## 5. Conclusions

Combinations of BiLdAg, LdSd and HaAa significantly weakened the overall contaminant degradation efficiency of mixed plant residue treatments. In particular, LdSd and HaAa mixtures simultaneously exerted remarkable inhibitory effects on the degradation of saturated hydrocarbons and aromatic hydrocarbon mixtures, including LdAgHa, BiLdAg, LdSd, LdAgHaAa, LdHa and HaAgAcSd, which significantly impaired the capacity of plant residues to replenish soil available nutrients and activate soil enzyme activities. The LdAgHa mixture exhibited the strongest antagonistic effects on the improvement efficiency of soil nitrate nitrogen content and sucrase, dehydrogenase and polyphenol oxidase activities (the increase relative to contaminated soil). Overall, the effects of mixed plant residue addition on contaminant degradation and the restoration of damaged soil biochemical properties were negatively correlated. Under experimental conditions, BiLdAg and LdSd residue combinations may lead to remarkable antagonistic interactions and lower the efficiency of necrophytoremediation, which should be avoided in practical field applications of this remediation technology.

## Figures and Tables

**Table 1:** Effects of crude oil contamination on the contents of available nutrients in soil. Different letters within the same column denote significant differences among litter treatments (*p* < 0.05).

Soil Samples	Nitrate Nitrogen (NO_3_-N) (mg·kg^−1^)	Ammonium Nitrogen (NH_4_-N) (mg·kg^−1^)	Available Phosphorus (P) (mg·kg^−1^)	Available Potassium (K) (mg·kg^−1^)
Uncontaminated	11.66 (0.11) a	11.34 (0.08) a	2.42 (0.21) a	70.50 (0.24) a
Crude oil contaminated	2.68 (0.03) b	6.81 (0.18) b	0.98 (0.35) b	65.00 (0.94) a
Natural attenuated	3.40 (0.12) b	2.36 (0.03) c	0.86 (0.00) b	62.50 (1.65) a

**Table 2:** Effects of crude oil contamination on soil enzyme activities. Different letters within the same column denote significant differences among litter treatments (*p* < 0.05).

Soil Samples	Sucrase (mg Glucose·g^−1^·h^−1^)	Urease (mg NH_2_-N·g^−1^·h^−1^)	Alkine Phosphatase (mg Phenols·g^−1^·h^−1^)	Catalase (mL 0.01 mol·L^−1^ KMnO_4_·g^−1^·h^−1^)	Dehydrogenase (mg Triphenylformazan·g^−1^·h^−1^)	Polyphenol Oxidase (mg Purpurogallin·g^−1^·h^−1^)
Uncontaminated	6.46 (0.63) a	0.08 (0.00) a	0.02 (0.00) c	3.76 (0.13) ab	0.15 (0.00) a	1.08 (0.01) a
Crude oil contaminated	3.80 (0.40) a	0.05 (0.00) b	0.07 (0.01) a	3.48 (0.20) b	0.22 (0.01) a	1.33 (0.05) a
Natural attenuated	3.99 (0.27) a	0.02 (0.00) b	0.05 (0.00) b	4.44 (0.30) a	0.17 (0.01) a	1.20 (0.08) a

**Table 3:** Initial chemical traits of the tested plant residues. Data are presented as the mean ± SE; different letters indicate significant differences among plant residues according to one-way analysis of variance (ANOVA), and Tukey’s HSD method was used for post hoc analysis, *p* < 0.05. Sd: *Sophora davidii*; Ag: *Artemisia gmelinii*; Aa: *Artemisia annua*; Bi: *Bothriochloa ischaemum*; Ld: *Lespedeza davurica*; Ha: *Heteropappus altaicus*; Ac: *Agropyron cristatum*. MFAD, FDiv and FSpe represent the modified functional attribute diversity, functional divergence, and functional specialization indices, respectively, of mixed plant residues.

Plant Residues	C c/(mg/g)	N c/(mg/g)	P c/(mg/g)	K c/(mg/g)	Ratio of C/N	Ratio of C/P	Ratio of N/P	Polyphenols c/(mg/g)	Soluble Sugars c/(mg/g)	Terpenoids c/(mg/g)	Amino Acids c/(μg/g)	Flavonoids c/(mg/g)	Organic Acids c/(μg/g)	MFAD	FDiv	FSpe
BiLdAg	352.66 (2.63) b	14.71 (0.12) f	1.33 (0.20) b	12.97 (0.00) g	26.86 (0.33) g	538.66 (48.87) a	17.30 (1.72) a	11.97 (0.23) e	1.02 (0.02) ef	12.39 (1.19) bc	7.50 (0.30) g	22.57 (0.80) g	9.44 (0.11) a	0.66 (0.02) b	1.6 (0.18) abc	2.51 (0.34) a
LdAgHa	297.35 (1.71) d	17.33 (0.26) e	1.71 (0.22) ab	16.48 (0.28) e	17.37 (0.18) e	287.19 (41.49) c	13.75 (1.69) ab	16.65 (0.33) d	1.24 (0.04) d	17.72 (1.68) a	8.42 (0.12) f	42.44 (0.92) e	8.58 (0.04) bc	0.63 (0.02) b	2.25 (0.48) a	2.49 (0.33) a
LdAgHaAa	322.69 (3.92) c	19.28 (0.27) d	2.02 (0.16) ab	18.58 (0.21) d	16.99 (0.13) d	249.02 (30.36) c	12.43 (1.22) bc	18.39 (0.38) c	1.22 (0.04) de	17.65 (1.57) a	12.22 (0.17) d	64.51 (1.09) d	8.62 (0.04) bc	0.95 (0.04) a	0.95 (0.07) c	1.04 (0.06) b
LdHa	220.70 (2.04) e	17.69 (0.34) e	2.19 (0.27) a	14.98 (0.28) f	12.49 (0.18) f	112.01 (9.51) e	8.88 (0.64) c	16.76 (0.46) d	1.49 (0.05) c	15.62 (1.51) ab	11.05 (0.00) e	42.47 (1.28) e	8.90 (0.09) b	0.35 (0.01) c	1.42 (0.37) abc	1.8 (0.25) ab
LdSd	310.90 (5.33) cd	23.57 (0.23) a	1.96 (0.19) ab	9.99 (0.00) h	12.97 (0.10) h	225.56 (11.45) cd	16.65 (0.61) a	10.77 (0.38) f	4.10 (0.15) a	8.06 (1.65) c	18.32 (0.12) a	12.51 (1.12) h	5.54 (0.10) e	0.36 (0.01) c	1.71 (0.4) abc	1.8 (0.38) ab
AgHaAa	357.58 (4.92) b	19.67 (0.22) cd	1.76 (0.05) ab	22.29 (0.19) b	18.65 (0.21) b	304.8 (36.35) bc	14.31 (1.28) ab	19.81 (0.30) b	0.99 (0.05) f	19.79 (2.09) a	12.94 (0.26) c	80.95 (1.82) c	8.20 (0.08) d	0.65 (0.00) b	1.97 (0.64) ab	2.53 (0.49) a
AgAa	424.67 (8.23) a	20.87 (0.19) b	1.86 (0.05) ab	22.19 (0.15) b	21.5 (0.28) b	386.03 (51.28) b	15.97 (1.80) ab	20.02 (0.42) b	0.94 (0.07) f	19.68 (1.66) a	13.38 (0.35) c	86.56 (0.98) b	8.33 (0.01) cd	0.35 (0.02) c	1.13 (0.13) bc	1.8 (0.21) ab
HaAa	311.04 (8.09) cd	21.19 (0.28) b	2.27 (0.02) a	23.69 (0.14) a	14.41 (0.31) a	138.43 (2.91) de	9.73 (0.03) c	21.5 (0.50) a	1.11 (0.06) def	18.71 (1.80) a	17.83 (0.20) a	100.24 (3.19) a	8.33 (0.26) cd	0.35 (0.02) c	1.68 (0.04) abc	1.85 (0.07) ab
HaAgAcSd	324.82 (0.50) c	20.02 (0.23) c	1.72 (0.04) ab	19.72 (0.15) c	16.73 (0.21) c	303.46 (20.01) bc	16.49 (0.47) a	11.99 (0.28) e	2.17 (0.09) b	15.8 (1.23) ab	16.60 (0.09) b	32.06 (1.70) f	5.79 (0.03) e	0.95 (0.02) a	0.91 (0.03) c	0.99 (0.03) b

## Data Availability

The original contributions presented in the study are included in the article; further inquiries can be directed to the corresponding authors.

## Supplementary Materials

The following supporting information can be downloaded at: https://www.mdpi.com/article/10.3390/plants15152263/s1, Table S1: Initial chemical traits of the tested monospecific plant residue.
