# Supplementary material for: Inappropriate Mixed Addition of Plant Residues Weakens the Remediation Effect of Crude Oil-Contaminated Soil
Source: Plants (Basel). 2026 Jul 24;15(15):2263. doi: 10.3390/plants15152263 (PMC13467582; doi:10.3390/plants15152263)
Supplement: Supplementary file 1 [file plants-15-02263-s001.zip › plants-4351661-supplementary.pdf]

plants

MDPI

Article

Inappropriate Mixed Addition of Plant Residues Weakens the Remediation Effect of Crude Oil-Contaminated Soil

Hongyan Hui [1], Liping Li [1], Xianyang Pan [1], Jing Xie [1], Xin Liu [1], Wenqing Jiang [1], Xincheng Huang [1], Xiaoxi Zhang [1,2,*] and Bo Liu [1,*]

Table S1. Initial chemical traits of the tested monospecific plant residue. Data are presented as the mean ± SE; different letters indicate significant differences among plant residues according to one-way analysis of variance (ANOVA), and Tukey's HSD method was used for post hoc analysis, $P < 0.05$. Sd: *Sophora davidii*; Ag: *Artemisia gmelinii*; Aa: *Artemisia annua*; Bi: *Bothriochloa ischaemum*; Ld: *Lespedeza davurica*; Ha: *Heteropappus altaicus*; Ac: *Agropyron cristatum*.

Plant residues	C c/(mg/g)	N c/(mg/g)	P c/(mg/g)	K c/(mg/g)	Ratio of C/N	Ratio of C/P	Ratio of N/P	Polyphenols c/(mg/g)	Soluble sugars c(mg/g)	Terpenoids c(mg/g)	Amino acids c(μg/g)	Flavonoids c(mg/g)	Organic acids c(μg/g)
Bi	389.32(13.79)b	9.39(0.13)d	0.43(0.00)d	11.96(0.57)d	41.44(1.07)a	896.77(32.38)a	21.63(0.32)a	5.35(0.20)d	0.41(0.05)c	3.99(0.11)c	9.29(0.60)de	10.11(0.70)d	10.50(0.00)a
Ld	218.01(5.37)c	18.12(0.42)c	2.81(0.48)b	7.48(0.29)e	12.03(0.11)d	81.69(12.41)d	6.79(1.05)b	14.13(0.67)c	1.90(0.01)b	11.24(4.12)bc	10.04(0.09)cd	15.21(1.11)d	9.90(0.10)a
Ag	450.65(1.46)a	16.62(0.08)c	0.75(0.12)cd	19.48(0.28)c	27.12(0.17)b	637.54(105.52)b	23.47(3.78)a	16.43(0.17)bc	0.74(0.04)c	21.93(2.77)a	3.15(0.38)e	42.37(1.70)c	7.90(0.30)ab
Ha	223.39(2.30)c	17.26(0.27)c	1.58(0.06)c	22.48(0.27)b	12.95(0.33)d	142.33(6.66)d	10.98(0.24)b	19.38(0.28)b	1.09(0.09)c	20.00(3.80)ab	12.06(0.08)c	69.73(3.67)b	7.90(0.30)ab
Sd	403.78(5.39)ab	29.02(0.05)a	1.11(0.09)cd	12.50(0.29)d	13.91(0.17)cd	369.44(35.01)c	26.51(2.21)a	7.41(1.02)d	6.29(0.31)a	4.88(0.82)c	26.60(0.15)a	9.81(1.80)d	1.20(0.10)c
Aa	398.69(17.84)b	25.12(0.29)b	2.97(0.03)b	24.90(0.01)a	15.87(0.65)c	134.53(6.73)d	8.47(0.18)b	23.61(0.98)a	1.14(0.11)c	17.43(2.30)ab	23.61(0.31)a	130.75(3.39)a	8.70(0.30)ab
Ac	221.45(4.76)c	17.16(0.54)c	3.44(0.07)a	24.44(0.32)ab	12.94(0.59)d	64.52(2.20)d	4.99(0.06)b	4.74(0.07)d	0.56(0.01)c	16.39(2.41)ab	24.58(0.66)a	6.32(2.10)d	6.10(0.10)b
Ac	221.45(4.76)c	17.16(0.54)c	3.44(0.07)a	24.44(0.32)ab	12.94(0.59)d	64.52(2.20)d	4.99(0.06)b	4.74(0.07)d	0.56(0.01)c	16.39(2.41)ab	24.58(0.66)a	6.32(2.10)d	6.10(0.10)b
